# Monocyte and Macrophage-Mediated Pathology and Protective Immunity During Schistosomiasis

**DOI:** 10.3389/fmicb.2020.01973

**Published:** 2020-08-12

**Authors:** Camila Oliveira Silva Souza, Luiz Gustavo Gardinassi, Vanderlei Rodrigues, Lúcia Helena Faccioli

**Affiliations:** ^1^Departamento de Análises Clínicas, Toxicológicas e Bromatológicas, Faculdade de Ciências Farmacêuticas de Ribeirão Preto, Universidade de São Paulo, São Paulo, Brazil; ^2^Departamento de Biociências e Tecnologia, Instituto de Patologia Tropical e Saúde Pública, Universidade Federal de Goiás, Goiânia, Brazil; ^3^Departamento de Bioquímica e Imunologia, Faculdade de Medicina de Ribeirão Preto, Universidade de São Paulo, São Paulo, Brazil

**Keywords:** monocyte, alternatively activated macrophage, IL-4Rα, fibrosis, transcriptomics, schistosomiasis

## Abstract

Infection by *Schistosoma* parasites culminates in a chronic granulomatous disease characterized by intense tissue fibrosis. Along the course of schistosomiasis, diverse leukocytes are recruited for inflammatory foci. Innate immune cell accumulation in Th2-driven granulomas around *Schistosoma* eggs is associated with increased collagen deposition, while monocytes and macrophages exert critical roles during this process. Monocytes are recruited to damaged tissues from blood, produce TGF-β and differentiate into monocyte-derived macrophages (MDMs), which become alternatively activated by IL-4/IL-13 signaling via IL-4Rα (AAMs). AAMs are key players of tissue repair and wound healing in response to *Schistosoma* infection. Alternative activation of macrophages is characterized by the activation of STAT6 that coordinates the transcription of *Arg1, Chi3l3, Relma*, and *Mrc1.* In addition to these markers, monocyte-derived AAMs also express *Raldh2* and *Pdl2.* AAMs produce high levels of IL-10 and TGF-β that minimizes tissue damage caused by *Schistosoma* egg accumulation in tissues. In this review, we provide support to previous findings about the host response to *Schistosoma* infection reusing public transcriptome data. Importantly, we discuss the role of monocytes and macrophages with emphasis on the mechanisms of alternative macrophage activation during schistosomiasis.

## Introduction

Schistosomiasis is a chronic helminthic disease caused by worms of the genus *Schistosoma* spp. (McManus et al., 2018). Poor and rural communities localized in tropical and sub-tropical regions are the most affected, where schistosomiasis causes ∼207,000 fatal outcomes every year (Schistosomiasis, 2020; World Health Organization [WHO], 2020 | Epidemiological situation). The three major species infect humans: *S. mansoni* (Africa and South America) and *S. japonicum* (East Asia), which mainly induce inflammation and fibrosis in intestine and liver; and *S. haematobium* (Africa and Middle East), which triggers urogenital manifestations (Gray et al., 2011).

The life cycle of *Schistosoma* involves an intermediate snail host and definitive mammalian hosts. Briefly, eggs released in the feces of a contaminated host reach an aquatic environment where the hatching miracidia from different species are attracted to snails of different genus such as *Biomphalaria, Oncomelania*, or *Bulinus*. After several morphological and molecular changes, mature cercariae exit the snail and swim at the surface until they actively penetrate into the host’s skin (Paveley et al., 2009; Collins et al., 2011). Within hours of penetration, the cercariae differentiate into the schistosomula, which reside in the skin for at least 2 days before migrating through blood vessels. Around 3–7 days, the schistosomula reach the lung, from where they are delivered to the hepatic portal circulation (Wilson, 2009; Schwartz et al., 2018). They mature into adult worms, mate, and later migrate to mesenteric venules (*S. mansoni and S. japonicum*) or bladder venules (*S. haematobium*) where they lay eggs. Hosts excrete eggs in the feces or urine around 6–7 weeks of infection (Schwartz and Fallon, 2018; Nation et al., 2020).

Host response to *Schistosoma* infection initiates with the activation of immune cells, which produce tumor necrosis factor (TNF)-α, interleukin (IL)-1β, IL-6, IL-12, and IFN-γ that promote an early Th1 response (Pearce et al., 1991; de Jesus et al., 2002; Mountford and Trottein, 2004). This profile changes dramatically with egg deposition (Pearce et al., 1991), whose accumulation into tissues induces Th2-driven granuloma, a hallmark of chronic disease (Pearce and MacDonald, 2002). Granulomas are structures composed of neutrophils, eosinophils, basophils, monocytes, macrophages (and their derivatives epithelioid and giants cells), T cells, B cells, and fibroblasts, which are recruited to limit tissue damage caused by eggs (Schwartz and Fallon, 2018).

Monocytes and macrophages exert important roles during schistosomiasis, while high levels of Th2 cytokines (IL-4 and IL-13) have profound phenotypical and functional impact on these cells (Pearce and MacDonald, 2002; McManus et al., 2018). In this review, we provide: (i) an overview of immune response dynamics; (ii) a discussion about monocytes and macrophages; and (iii) insights into the alternative activation of macrophages during schistosomiasis. To support the discussion, we reused public transcriptomes of liver from mice infected with *S. japonicum* (Burke et al., 2010).

## Brief Overview of the Immune Response to Schistosoma Infection

Penetration of cercariae into host’s skin induces the influx of neutrophils (Paveley et al., 2009), as well as the activation of resident macrophages, Langerhans cells (Kumkate et al., 2007) and dendritic cells (DCs) (Winkel et al., 2018). Upon activation by *S. mansoni* cercariae, resident macrophages release IL-10, while DCs produce IL-6, IL-12p40, TNF-α to activate the adaptive immune response in skin-draining lymph nodes (sdLN) (Mountford and Trottein, 2004; Paveley et al., 2009). For that, DCs increase the expression of HLA-DR, CD80, CD86, PDL-1, and PD-L2, interact with T cells and coordinate Th2 polarization (Winkel et al., 2018). Moreover, cercariae antigens are internalized by macrophages and influence CD4^+^IL-4^+^ T cells responses in sdLN (Paveley et al., 2011). Interestingly, multiple exposures (4x) to *S. mansoni* increases alternatively activated macrophage-like cells in the skin of mice and renders T cells hypo-responsive in an IL-4Rα-dependent manner (Cook et al., 2011). In addition, radiation-attenuated (RA) larvae of *S. mansoni* induces IL-12 production by CD11c^+^, F4/80^+^ skin-cells that drives a protective response (Hogg et al., 2003).

The cercariae that transform into schistosomula migrate through skin and enter the circulation (Schwartz et al., 2018). Anti-helminthic effectors are activated to kill migrating schistosomula, while the lung microenvironment constitutes an effective site to larvae elimination (von Lichtenberg et al., 1977; Wilson, 2009; Schwartz et al., 2018). Macrophages and eosinophils expressing FcεRI recognize IgE and induce antibody dependent cell mediated cytotoxicity (ADCC) toward larvae (Dombrowicz et al., 2000). Immunization with Sm-p80 antigen (*S. mansoni*) induces effective ADCC in lung during larvae migration (Torben et al., 2012), while SjCL3 (*S. japonicum*) suppresses the ADCC to evade the host response (Huang et al., 2020). Interestingly, TNF-α plays a significant role for innate lung immunity after RA-larvae vaccination, as TNFRI deficiency abrogates protective immunity, including IgG responses (Street et al., 1999).

**FIGURE 1:**
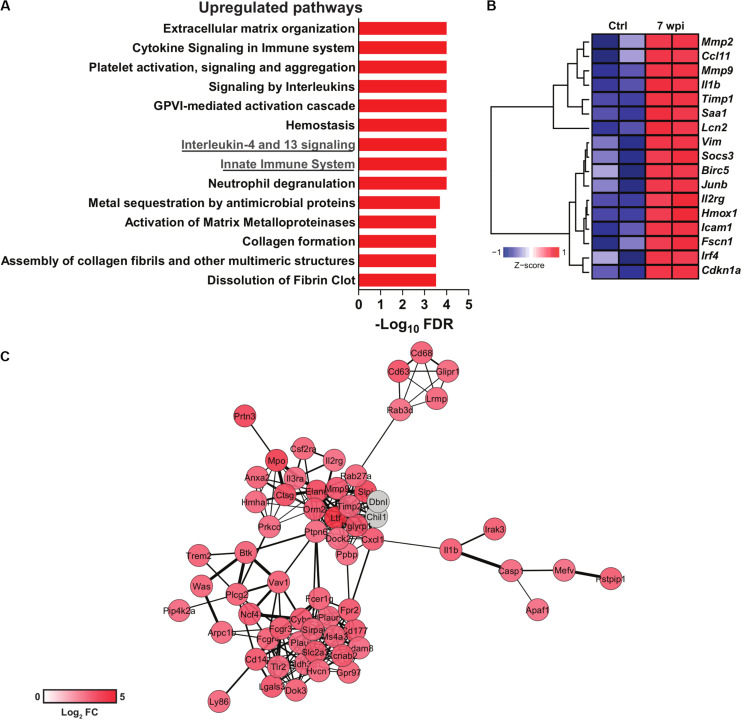
Transcriptomics data reuse of mice infected with *S. japonicum*. Normalized gene expression data from livers of mice infected with *S. japonicum* for 7 weeks and naïve mice were acquired at the Gene Expression Omnibus (GEO) repository (GSE14367). Differential gene expression was evaluated with the package *limma* for R. **(A)** Pathway enrichment analysis of upregulated genes in liver of *S. japonicum-*infected mice. Pathways were evaluated with the ToppGene suite platform (Chen et al., 2009) and the bar plot depicts the significance of enrichment in each pathway as shown by -log_10_ *p*-values adjusted by Benjamini-Hochberg false discovery rate (FDR) method. **(B)** Heat map showing the expression of genes included in the interleukin-4 and 13 signaling pathway in *S. japonicum*-infected and naïve mice. Hierarchical clustering was performed with the package *amap* for R using Euclidian distance metric and Ward linkage. The heat map was generated with the package *gplots* for R. **(C)** Gene network composed of genes included in the innate immune system pathway. The network was generated with the platform STRING v11.0 (Szklarczyk et al., 2015) and visualized with Cytoscape v3.7.2 (Shannon et al., 2003). The color scale is proportional to the log_2_ fold changes of each gene from *S. japonicum*-infected mice over naïve mice. Genes colored in gray were imputed by STRING but were not present in the evaluated dataset.

As the surviving schistosomula end up as adults in the intestine or bladder, the host adapts to the challenges imposed by adult worm burden and egg deposition. *S. mansoni*-derived lysophosphatidylcholine (LPC) activates eosinophils via TLR2 (Magalhães et al., 2010). Metalloproteinases (MMP), histamines and collagenases released by eosinophils contribute to tissue remodeling (Ariyaratne and Finney, 2019), while macrophage migration inhibitory factor (MIF) boosts IL-5 production and eosinophilia during schistosomiasis (Magalhães et al., 2009). Interestingly, *S. japonicum* induces more neutrophil influx into hepatic granulomas when compared to *S. mansoni* infection, which induces more eosinophils (Schwartz and Fallon, 2018). Upregulated neutrophil degranulation pathway in transcriptomics data support these findings (Figure 1A). In addition, *S. japonicum* eggs induce neutrophil extracellular traps, MMP9, IL-1β, IL-1α, CCL3, and CXCL2, which precedes granuloma formation (Chuah et al., 2014).

*Schistosoma* infection induces the release of the alarmins IL-33 (Peng et al., 2016; Li et al., 2019), TSLP and IL-25 (Cook et al., 2011; Vannella et al., 2016). IL-33 promotes the development of CD4^+^ IL-5^+^; IL-10^+^, and IL-13^+^ Th2 cells (Yu et al., 2015). During *S. japonicum* infection, IL-33 expression was associated with levels of tissue transglutaminase (tTG) protein (Li et al., 2019), while the numbers of macrophages expressing the IL-33 receptor (ST2L) were also increased when compared to CD3^+^ST2L^+^ T cells (Peng et al., 2016). Hepatic fibrosis induced by *S. mansoni* is not affected by the lack of TSLP, IL-25, and IL-33 signaling individually, but disruption of signaling by all three mediators reduced hepatic fibrosis, eosinophils, and innate lymphoid cell (ILC)-2 in the liver of mice (Vannella et al., 2016). Compared to CD4^+^ T cells, ILC2s produce high levels of IL-13, but not IL-4, while IL-25 triggers IL-13^+^ ILC2-mediated fibrosis in the lung after injection of *Schistosoma* eggs (Hams et al., 2014).

Signaling by IL-13 and IL-4 has been associate with the severity of schistosomiasis and fibrosis. Indeed, high levels of IL-13 in chronic schistosomiasis patients with hepatic fibrosis correlates with progression of the disease (Mutengo et al., 2018). Polymorphisms in IL-4 and STAT6 promoter gene were associated higher susceptibility to *S. haematobium* infection in children (Adedokun et al., 2018). Also, IL-4Rα signaling was associated with granuloma formation and bladder pathogenesis after injection of *S. haematobium* eggs (Mbanefo et al., 2020). Interestingly, 5-lipoxygenase metabolites induce IL-4 and IL-13 that control granuloma size in lung of mice injected with *S. mansoni* eggs (Toffoli da Silva et al., 2016). Indeed, *S. japonicum* infection upregulates the IL-4 and IL-13 signaling pathway in the murine liver (Figure 1A), which includes genes related to extracellular matrix remodeling such as *Mmp2* and *Mmp9* (Figure 1B). Strikingly, IL-4Rα deficient mice vaccinated with RA-attenuated larvae exhibit impaired production of protective IgG1 and IgE antibodies and cytokines such as IL-10 and IL-13 (Mountford et al., 2001). Indeed, the absence of IL-4Rα in B cells affects both cellular and humoral Th2 responses, increasing the susceptibility to *S. mansoni* infection (Hurdayal et al., 2017).

Hepatic inflammation during *S. japonicum* infection initiates with the recruitment of CD4^+^ T; CD8^+^ T cells and CD19^+^ B cells, whereas eosinophil, neutrophils and F4/80^+^CD11b^+^ cells increase at 7 wpi (Burke et al., 2010). Curiously, the *S. haematobium* bladder-granuloma are dominated by CD68^+^cells (syncytial macrophages) (Fu et al., 2012). The peak of collagen deposition correlates with MIP-1α and CXCL1 production in *S. haematobium* infection (Fu et al., 2012), and innate immune cell influx to the hepatic granuloma in *S. japonicum* infection (Burke et al., 2010). Innate immune cell activation is reflected by a robust upregulation of genes composing an innate immunity network (Figure 1C). Monocytes and macrophages play critical roles for the granuloma formation and immune regulation. Many molecules and mechanisms shape their phenotypes and functions along *Schistosoma* infection and will be discussed in the next sections.

## Origin and Function of Monocytes During Schistosomiasis

**FIGURE 2:**
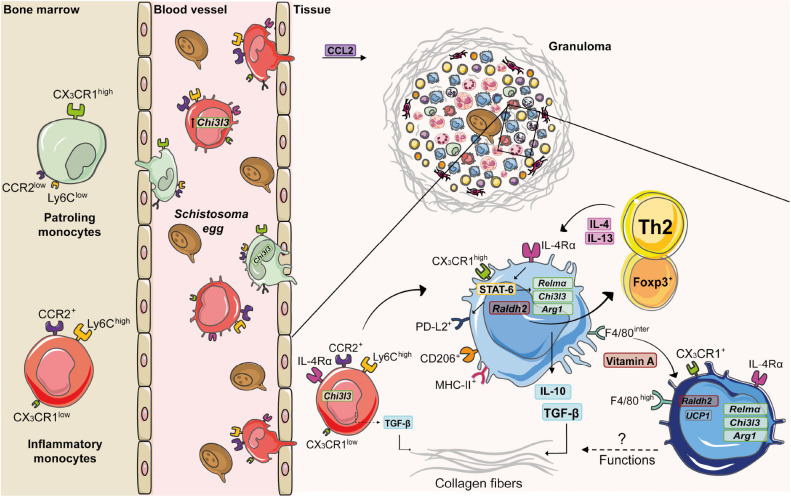
Monocyte and macrophage dynamics during experimental *S. mansoni* infection. Along the course of experimental *S. mansoni* infection in mice, bone marrow increases the monopoiesis and originates two subsets: inflammatory monocytes (Ly6C^high^CCR2^high^CX_3_CR1^low^) and patrolling monocytes (Ly6C^low^CCR2^low^CX_3_CR1^high^), which are recruited from blood to tissues affected by egg accumulation. Upon activation by circulating eggs, inflammatory monocytes express higher levels of chitinase-like 3 (*Chi3l3*) compared to patrolling monocytes. CCL2 mediates inflammatory monocyte recruitment to the liver during schistosomiasis. Inside the tissue, inflammatory monocytes produce TGF-β, induce collagen deposition and differentiate into macrophages. The diversity of cells present in the granuloma is responsible for a type 2 microenvironment, whereby T cells produce IL-4 and IL-13 that induces the alternative activation of macrophages via IL-4Rα. The IL-4/IL-13/IL-4Rα axis leads to the transcription of retinaldehyde dehydrogenase 2 (*Raldh2)* and activation of signal transducer and activator of transcription 6 (STAT-6). This transcription factor upregulates the expression of *Chi3l3*, programmed cell death ligand 2 (*Pdl2)*, arginase -1 (*Arg1)* and resistin-like alpha *(Relma)*, which trigger TGF-β production and collagen deposition. RALDH2 by AAM induces Treg cell differentiation. Vitamin A mediates conversion of monocyte-derived macrophages (F4/80^int^CD206^+^PD-L2^+^MHC-II^+^) into F4/80^high^CD206^–^ PD-L2^–^ MHC-II^–^ UCP1^+^ phenotype, whose function still needs to be elucidated.

Monocytes play important roles in homeostasis, host defense, resolution of inflammation and tissue repair (Hume et al., 2019). They first originate during embryogenesis, seed diverse organs and develop into resident macrophage populations, such as alveolar macrophages in the lung and Kupffer cells in the liver (Zhao et al., 2018). After birth, progenitor cells in the bone marrow and spleen ultimately differentiate into the stage “*common Monocytes Progenitor – cMoP*” [lineage-negative (Lin^–^) CD117^+^ CD115^+^ CD125^–^ Ly6C^+^ CD11b^–^], which further transform into distinct subsets (Hettinger et al., 2013). Based on the differential expression of surface markers, monocytes are classified as: inflammatory monocytes (classical) (mouse: Ly6C^high^CCR2^+^CX_3_CR1^low^; human: CD14^high^CD16^–^), which are robustly recruited to sites of inflammation (Geissmann et al., 2003), but return to the bone marrow after 3 days under steady state (Varol et al., 2007); and patrolling monocytes (non-classical) (mouse: Ly6C^low^CCR2^low^CX_3_CR1^high;^ human: CD14^low/–^CD16^high^), which patrol blood vessels and remain in the circulation for as long as 7 days (Auffray et al., 2007; Figure 2). Recently, we showed that low expression of CD18 (common subunit of β2 integrins) was associated with a reduction of monocyte subsets in bone marrow and blood of mice infected with *S. mansoni* (Souza et al., 2018). This correlated with increased parasite burden, egg deposition and mortality of mice.

Monocytes promote resistance to schistosomiasis (Nascimento et al., 2014; Souza et al., 2018; de Souza et al., 2019). Indeed, inflammatory monocyte depletion causes a severe weight loss and disrupts a protective Th2 response during *S. mansoni* infection (Nascimento et al., 2014). Intermediate human monocytes (CD14^bright^CD16^+^) from schistosomiasis patients bind cercariae E/S ligands more efficiently than those from healthy individuals, indicating a role for monocytes in parasite recognition (Turner et al., 2014). In addition, adoptive transference of CD11b^+^CD14^+^ bone marrow cells to mice infected with *S. mansoni* reduced the expression of TGF-β1 and collagen deposition in the liver (de Souza et al., 2019).

Despite of their protective functions, monocytes also induce fibrosis (Fernandes et al., 2014). For example, B1 cells secrete IL-10 after *S. japonicum* infection and inhibit CCL2 chemokine (ligand CCR2) production, leading to reduced Ly6C^high^ monocytes and fibrosis in the liver (Yong et al., 2019). Moreover, CD14^high^CD16^–^ and CD14^low/–^CD16^high^ monocytes from schistosomiasis patients with liver fibrosis increase the expression of TGF-β (Fernandes et al., 2014). In addition, stimulation of monocytes with soluble egg antigen (SEA) upregulates the expression of TGF-β (Wolde et al., 2020; Figure 2). Curiously, lack of CX_3_CR1 and thus, patrolling monocytes, reduced liver weights and granuloma formation in mice infected with *S. japonicum*, suggesting a differential role for monocyte subsets in this model (Ran et al., 2015).

## The Impact of Alternative Macrophage Activation on Schistosoma Infection

Resident macrophages localize in different tissues to maintain the homeostasis (Zhao et al., 2018) and exhibit particular phenotypes and molecular programs (Coakley and Harris, 2020). During schistosomiasis, most macrophages in inflammatory sites are derived from blood monocytes (Rückerl and Cook, 2019). Depending on environmental cues, monocytes differentiate into macrophages that assume a spectrum of molecular profiles (Das et al., 2015). Two of these molecular programs have been extensively studied: the classically activated macrophages (M1), which are induced by IFN-γ + LPS and the alternatively activated macrophages (AAMs/M2), which are induced by IL-4 + IL-13 (Martinez and Gordon, 2014). Many studies show that IL-4/IL-13 signaling via IL-4Rα activates the transcription factor signal transducer and activator of transcription 6 (STAT-6), which upregulates the expression of arginase -1 (*Arg1*), chitinase-like 3 (*Chi3l3*), resistin-like alpha (*Relma*), and mannose receptor C-type 1 (*Mrc1*) (Gordon, 2003; Gordon and Martinez, 2010; Rolot and Dewals, 2018). Although IL-4Rα signaling induces alternative phenotype in both tissue resident macrophages (Jenkins et al., 2011) and MDMs during helminth infections (Gundra et al., 2014; Rolot et al., 2019), they have distinct transcriptional signatures, phenotypes and functions. Both resident and monocyte-derived AAMs express high levels of *Arg1*, *Chi3l3*, and *Relma*, but only monocyte-derived AAMs upregulate the expression of retinaldehyde dehydrogenase 2 (*Raldh2)* and programmed cell death ligand 2 (*Pdl2)* (Gundra et al., 2014).

The influx of blood monocytes and their conversion to macrophages occurs independently of IL-4Rα during *S. mansoni* infection (Rolot et al., 2019). The Th2 environment developed in response to *Schistosoma* eggs provides signals for AAM polarization (Figure 2). For example, basophils sense the egg glycoprotein IPSE/alpha-1, produce IL-4 and IL-13 and trigger the alternative activation of human monocytes, demonstrated by the increased expression of CD206^+^ and CD209^+^ (Knuhr et al., 2018). In addition, chronic *S. mansoni* infection induces Ly6C^high^ monocyte migration into the liver, which differentiate into CD206^+^PD-L2^+^ AAMs in a CD4^+^ T cell dependent manner (Girgis et al., 2014). Macrophages stimulated with *Sm16* antigen induce high levels of IL-10 that block the M1 activation in response to IFN-γ and LPS (Sanin and Mountford, 2015). Interestingly, SEA induces the expression of M2 markers (*Arg1*, CD206, IL-10, and TGF-β) and higher STAT-6 phosphorylation in J774A.1 macrophage cell line (Tang et al., 2017). Moreover, *S. mansoni*’s LPC induces higher expression of arginase-1 and CD206, while increasing the production of IL-10, TGF-β, and prostaglandin E_2_ by peritoneal macrophages (PMs) *in vitro* (Assunção et al., 2017).

Macrophages are protagonists of tissue repair during schistosomiasis (Herbert et al., 2004; Borthwick et al., 2016). IL-4Rα signaling in AAMs induces the production of IL-10 and TGF-β, promoting healing that minimizes collateral tissue damage during the granuloma formation (Barron and Wynn, 2011). Upon *S. mansoni* infection, *LysM^*cre*^IL-4R*α*^–/flox^* mice fail to mount an effective Th2 immune response, resulting in liver damage and increased mortality due high levels of IFN-γ production (Herbert et al., 2004). Importantly, depletion of F4/80^+^CD11b^+^ macrophages after the injection of *S. mansoni* eggs reduced the size of granulomas, downregulated the expression of *IL-13ra2* (IL-13Rα2), *Retnla* and *Chi3l3* and affected CD4^+^ Th2 cell responses in the lung (Borthwick et al., 2016). Interestingly, IL-4Rα-dependent AAM polarization mediates sdLN CD4^+^T cell hypo-responsiveness to repeated exposure to cercariae via IL-10 production (Prendergast et al., 2016). Macrophages are crucial for eosinophil, T cell and B cell recruitment into granuloma and orchestrate the response to preserve the bladder integrity after *S. haematobium* infection (Fu et al., 2015). The protective role of macrophages during *S. haematobium* infection was correlated with increased expression of *Il4*, *Arg1*, MMP, and collagenase genes in the bladder (Ray et al., 2012).

Molecules that characterize AAMs exhibit diverse functions and have been associated with protection or immunopathology during schistosomiasis. For example, injection of *S. mansoni* eggs in *Retnla*^–/–^ mice induces increased pulmonary inflammation and disorganized collagen-fiber deposition around granulomas (Nair et al., 2009). Upon infection with *S. mansoni*, *Arg1*^–^*^/^*^–^ mice produce less IL-10 and TGF-β, lose more weigh, show increased intestinal egg burden and present higher mortality rates (Herbert et al., 2010). Importantly, *LsyM^*cre*^Arg^–/flox^* mice are highly susceptible to *S. mansoni* infection and exhibit exacerbated granulomatous inflammation, liver fibrosis, and portal hypertension. This is tightly connected to macrophage function, because AAM from *LsyM^*cre*^Arg^–/flox^* mice inhibit T cell proliferation, independently of IL-10 and TGF-β (Pesce et al., 2009). PMs from *S. japonicum-*infected mice increase the expression of CD206, CCL2, IL-10, and *Arg1* and reduce the expression of CD16/CD32 (M1), while stimulation with SEA also induced higher expression of CD206, IL-10 and *Arg1* in PMs (Zhu et al., 2014). Interestingly, upregulated the expression of *Chi3l3* and *Arg-1* in AAMs from *S. japonicum*-infected mice was associated with increased fibrosis and immunopathology in the liver (Ye et al., 2020). Of interest, IL-10 plays a key role in maintaining AAMs and control of liver damage in the absence of IL-4Rα signaling during *S. mansoni* infection (Dewals et al., 2010). IL-10 regulates anti-fibrotic processes (Kamdem et al., 2018), while low levels of IL-10 was associated with severe hepatic fibrosis during schistosomiasis patients (Mutengo et al., 2018). The cooperation of IL-10 production by macrophages and CD4^+^ T cells was associated with reduce morbidity to experimental schistosomiasis (Hesse et al., 2004). IL-10^–/–^ mice exhibit increased granuloma areas after *S. mansoni* infection (Sadler et al., 2003), but this cytokine is not able to compensate the damaging effects of the absence of IL-4Rα-dependent AAM activation (Herbert et al., 2004). In addition, vitamin A is also an important signal for AAM differentiation and polarization during schistosomiasis. Mice infected with *S. mansoni* increase the expression of *Raldh2* and *Raldh3* in the liver, whereas AAMs induce the differentiation of CD4^+^Foxp3^+^ regulatory T cells via retinoic acid metabolism. Of note, mice that lack vitamin A exhibit increased hepatic damage, failure to form granulomas (Broadhurst et al., 2012) and increased mortality in response to *S. mansoni* infection (Gundra et al., 2017). Indeed, vitamin A is required for the conversion of monocyte-derived F4/80^int^CD206^+^PD-L2^+^MHC-II^+^ macrophages into cells that assume a F4/80^high^CD206^–^PD-L2^–^MHC-II^–^UCP1^+^ phenotype (Gundra et al., 2017), whose function during infection and/or resolution still needs to be elucidated (Figure 2).

## Conclusion and Future Directions

In the last few years, there were several advances in the understanding about the relationship between monocytes/macrophages and the Th2 response during schistosomiasis. Along the progression of schistosomiasis, diverse cells are recruited for inflammatory foci in response to worms, eggs and antigens. Granuloma formation around eggs accumulating in tissues requires monocyte recruitment from blood, which differentiate into macrophages and polarize into an alternatively activated phenotype via IL-4Rα signaling. AAMs are key players of efficient tissue repair and wound healing in response to *Schistosoma* infection. However, deregulated responses may also result in pathogenic fibrosis, a clinical condition that have been associated with mortality due schistosomiasis (Andrade, 2009). Several factors contribute to monocyte differentiation into macrophages and the alternative activation of these phagocytes, including their metabolism. These processes have been extensively explored in diverse studies and coordinate cellular functions and phenotype. Therefore, pharmacological manipulation of monocyte/macrophage metabolism may offer interesting opportunities to design novel therapies for severe schistosomiasis.

## Author Contributions

CS reviewed the literature, conceptualized the figures, and wrote the manuscript. LG performed the data analysis and critically revised the manuscript. VR discussed and critically revised the manuscript. LF critically revised the manuscript and acquired funding. All authors read and approved the final version of the manuscript.

## Conflict of Interest

The authors declare that the research was conducted in the absence of any commercial or financial relationships that could be construed as a potential conflict of interest.

## References

[B1] Adedokun S. A. Seamans B. N. Cox N. T. Liou G. Akindele A. A. Li Y. (2018). Interleukin-4 and STAT6 promoter polymorphisms but not interleukin-10 or 13 are essential for schistosomiasis and associated disease burden among Nigerian children. *Infect. Genet. Evol.* 65 28–34. 10.1016/j.meegid.2018.07.012 30010060

[B2] Andrade Z. A. (2009). Schistosomiasis and liver fibrosis. *Parasite Immunol.* 31, 656–663. 10.1111/j.1365-3024.2009.01157.x 19825105

[B3] Ariyaratne A. Finney C. A. M. (2019). Eosinophils and macrophages within the Th2-induced granuloma: balancing killing and healing in a tight space. *Infect. Immun.* 87:e127-19 10.1128/IAI.00127-119 PMC6759305 31285249

[B4] Assunção L. S. Magalhães K. G. Carneiro A. B. Molinaro R. Almeida P. E. Atella G. C. (2017). Schistosomal-derived lysophosphatidylcholine triggers M2 polarization of macrophages through PPARγ dependent mechanisms. *Biochim. Biophys. Acta Mol. Cell Biol. Lipids* 1862 246–254. 10.1016/j.bbalip.2016.11.006 27871882

[B5] Auffray C. Fogg D. Garfa M. Elain G. Join-Lambert O. Kayal S. (2007). Monitoring of blood vessels and tissues by a population of monocytes with patrolling behavior. *Science* 317 666–670. 10.1126/science.1142883 17673663

[B6] Barron L. Wynn T. A. (2011). Macrophage activation governs schistosomiasis-induced inflammation and fibrosis. *Eur. J. Immunol.* 41 2509–2514. 10.1002/eji.201141869 21952807 PMC3408543

[B7] Borthwick L. A. Barron L. Hart K. M. Vannella K. M. Thompson R. W. Oland S. (2016). Macrophages are critical to the maintenance of IL-13-dependent lung inflammation and fibrosis. *Mucosal Immunol.* 9 38–55. 10.1038/mi.2015.34 25921340 PMC4626445

[B8] Broadhurst M. J. Leung J. M. Lim K. C. Girgis N. M. Gundra U. M. Fallon P. G. (2012). Upregulation of retinal dehydrogenase 2 in alternatively activated macrophages during retinoid-dependent Type-2 immunity to helminth infection in mice. *PLoS Pathog.* 8:2883. 10.1371/journal.ppat.1002883 22927819 PMC3426520

[B9] Burke M. L. McManus D. P. Ramm G. A. Duke M. Li Y. Jones M. K. (2010). Temporal expression of chemokines dictates the hepatic inflammatory infiltrate in a murine model of schistosomiasis. *PLoS Negl. Trop. Dis.* 4:e598. 10.1371/journal.pntd.0000598 20161726 PMC2817718

[B10] Chen J. Bardes E. E. Aronow B. J. Jegga A. G. (2009). ToppGene suite for gene list enrichment analysis and candidate gene prioritization. *Nucleic Acids Res.* 37 W305–W311. 10.1093/nar/gkp427 19465376 PMC2703978

[B11] Chuah C. Jones M. K. Burke M. L. McManus D. P. Owen H. C. Gobert G. N. (2014). Defining a pro-inflammatory neutrophil phenotype in response to schistosome eggs. *Cell. Microbiol.* 16 1666–1677. 10.1111/cmi.12316 24898449

[B12] Coakley G. Harris N. L. (2020). Interactions between macrophages and helminths. *Parasite Immunol.* 2020:e12717. 10.1111/pim.12717 32249432

[B13] Collins J. J. King R. S. Cogswell A. Williams D. L. Newmark P. A. (2011). An atlas for schistosoma mansoni organs and life-cycle stages using cell type-specific markers and confocal microscopy. *PLoS Negl. Trop. Dis.* 5:1009. 10.1371/journal.pntd.0001009 21408085 PMC3050934

[B14] Cook P. C. Aynsley S. A. Turner J. D. Jenkins G. R. Rooijen N. V. Leeto M. (2011). Multiple helminth infection of the skin causes lymphocyte hypo-responsiveness mediated by Th2 conditioning of dermal myeloid cells. *PLoS Pathog.* 7:e1001323. 10.1371/journal.ppat.1001323 21445234 PMC3060168

[B15] Das A. Sinha M. Datta S. Abas M. Chaffee S. Sen C. K. (2015). Monocyte and macrophage plasticity in tissue repair and regeneration. *Am. J. Pathol.* 185 2596–2606. 10.1016/j.ajpath.2015.06.001 26118749 PMC4607753

[B16] de Jesus A. R. Silva A. Santana L. B. Magalhães A. de Jesus A. A. de Almeida R. P. (2002). Clinical and immunologic evaluation of 31 patients with acute *Schistosomiasis mansoni*. *J. Infect. Dis.* 185 98–105. 10.1086/324668 11756987

[B17] de Souza V. C. A. Moura D. M. N. de Castro M. C. A. B. Bozza P. T. de Almeida Paiva L. Fernandes C. J. B. (2019). Adoptive transfer of bone marrow-derived monocytes ameliorates schistosoma mansoni -induced liver fibrosis in mice. *Sci. Rep.* 9:6434. 10.1038/s41598-019-42703-y 31015492 PMC6478942

[B18] Dewals B. G. Marillier R. G. Hoving J. C. Leeto M. Schwegmann A. Brombacher F. (2010). IL-4Rα-independent expression of mannose receptor and Ym1 by macrophages depends on their IL-10 responsiveness. *PLoS Negl. Trop. Dis.* 4:e689. 10.1371/journal.pntd.0000689 20502521 PMC2872644

[B19] Dombrowicz D. Quatannens B. Papin J. P. Capron A. Capron M. (2000). Expression of a functional Fc epsilon RI on rat eosinophils and macrophages. *J. Immunol.* 165 1266–1271. 10.4049/jimmunol.165.3.1266 10903725

[B20] Fernandes J. S. Araujo M. I. Lopes D. M. de Souza R. da P. Carvalho E. M. (2014). Monocyte subsets in schistosomiasis patients with periportal fibrosis. *Med. Inflamm.* 2014:703653. 10.1155/2014/703653 24757288 PMC3976880

[B21] Fu C.-L. Odegaard J. I. Herbert D. R. Hsieh M. H. (2012). A novel mouse model of Schistosoma haematobium egg-induced immunopathology. *PLoS Pathog.* 8:e1002605. 10.1371/journal.ppat.1002605 22479181 PMC3315496

[B22] Fu C.-L. Odegaard J. I. Hsieh M. H. (2015). Macrophages are required for host survival in experimental *Urogenital schistosomiasis*. *FASEB J.* 29 193–207. 10.1096/fj.14-259572 25351984 PMC4285549

[B23] Geissmann F. Jung S. Littman D. R. (2003). Blood monocytes consist of two principal subsets with distinct migratory properties. *Immunity* 19 71–82. 10.1016/s1074-7613(03)00174-2 12871640

[B24] Girgis N. M. Gundra U. M. Ward L. N. Cabrera M. Frevert U. Loke P. (2014). Ly6C(high) monocytes become alternatively activated macrophages in schistosome granulomas with help from CD4+ cells. *PLoS Pathog.* 10:e1004080. 10.1371/journal.ppat.1004080 24967715 PMC4072804

[B25] Gordon S. (2003). Alternative activation of macrophages. *Nat. Rev. Immunol.* 3 23–35. 10.1038/nri978 12511873

[B26] Gordon S. Martinez F. O. (2010). Alternative activation of macrophages: mechanism and functions. *Immunity* 32 593–604. 10.1016/j.immuni.2010.05.007 20510870

[B27] Gray D. J. Ross A. G. Li Y.-S. McManus D. P. (2011). Diagnosis and management of schistosomiasis. *BMJ* 342:d2651. 10.1136/bmj.d2651 21586478 PMC3230106

[B28] Gundra U. M. Girgis N. M. Gonzalez M. A. San Tang M. Van Der Zande H. J. P. Lin J.-D. (2017). Vitamin A mediates conversion of monocyte-derived macrophages into tissue-resident macrophages during alternative activation. *Nat. Immunol.* 18 642–653. 10.1038/ni.3734 28436955 PMC5475284

[B29] Gundra U. M. Girgis N. M. Ruckerl D. Jenkins S. Ward L. N. Kurtz Z. D. (2014). Alternatively activated macrophages derived from monocytes and tissue macrophages are phenotypically and functionally distinct. *Blood* 123 e110–e122. 10.1182/blood-2013-08-520619 24695852 PMC4023427

[B30] Hams E. Armstrong M. E. Barlow J. L. Saunders S. P. Schwartz C. Cooke G. (2014). IL-25 and type 2 innate lymphoid cells induce pulmonary fibrosis. *Proc. Natl. Acad. Sci. U.S.A.* 111 367–372. 10.1073/pnas.1315854111 24344271 PMC3890791

[B31] Herbert D. R. Hölscher C. Mohrs M. Arendse B. Schwegmann A. Radwanska M. (2004). Alternative macrophage activation is essential for survival during schistosomiasis and downmodulates T helper 1 responses and immunopathology. *Immunity* 20 623–635. 10.1016/s1074-7613(04)00107-104 15142530

[B32] Herbert D. R. Orekov T. Roloson A. Ilies M. Perkins C. O’Brien W. (2010). Arginase I suppresses IL-12/IL-23p40-driven intestinal inflammation during acute schistosomiasis. *J. Immunol.* 184 6438–6446. 10.4049/jimmunol.0902009 20483789 PMC2921223

[B33] Hesse M. Piccirillo C. A. Belkaid Y. Prufer J. Mentink-Kane M. Leusink M. (2004). The pathogenesis of schistosomiasis is controlled by cooperating IL-10-producing innate effector and regulatory T cells. *J. Immunol.* 172 3157–3166. 10.4049/jimmunol.172.5.3157 14978122

[B34] Hettinger J. Richards D. M. Hansson J. Barra M. M. Joschko A.-C. Krijgsveld J. (2013). Origin of monocytes and macrophages in a committed progenitor. *Nat. Immunol.* 14 821–830. 10.1038/ni.2638 23812096

[B35] Hogg K. G. Kumkate S. Anderson S. Mountford A. P. (2003). Interleukin-12 p40 Secretion by Cutaneous CD11c+ and F4/80+ cells is a major feature of the innate immune response in mice that develop Th1-mediated protective immunity to *Schistosoma mansoni*. *Infect. Immun.* 71 3563–3571. 10.1128/IAI.71.6.3563-3571.2003 12761141 PMC155763

[B36] Huang W. Gu M. Cheng W. Zhao Q. P. Ming Z. Dong H. (2020). Characteristics and function of cathepsin L3 from *Schistosoma japonicum*. *Parasitol. Res.* 119 1619–1628. 10.1007/s00436-020-06647-x 32185481

[B37] Hume D. A. Irvine K. M. Pridans C. (2019). The mononuclear phagocyte system: the relationship between monocytes and macrophages. *Trends Immunol.* 40 98–112. 10.1016/j.it.2018.11.007 30579704

[B38] Hurdayal R. Ndlovu H. H. Revaz-Breton M. Parihar S. P. Nono J. K. Govender M. (2017). IL-4-producing B cells regulate T helper cell dichotomy in type 1- and type 2-controlled diseases. *Proc. Natl. Acad. Sci. U.S.A.* 114 E8430–E8439. 10.1073/pnas.1708125114 28916732 PMC5635893

[B39] Jenkins S. J. Ruckerl D. Cook P. C. Jones L. H. Finkelman F. D. van Rooijen N. (2011). Local macrophage proliferation, rather than recruitment from the blood, is a signature of Th2 inflammation. *Science* 332 1284–1288. 10.1126/science.1204351 21566158 PMC3128495

[B40] Kamdem S. D. Moyou-Somo R. Brombacher F. Nono J. K. (2018). Host regulators of liver fibrosis during human *Schistosomiasis*. *Front. Immunol.* 9:2781. 10.3389/fimmu.2018.02781 30546364 PMC6279936

[B41] Knuhr K. Langhans K. Nyenhuis S. Viertmann K. Kildemoes A. M. O. Doenhoff M. J. (2018). Schistosoma mansoni Egg-released IPSE/alpha-1 dampens inflammatory cytokine responses via basophil interleukin (IL)-4 and IL-13. *Front. Immunol.* 9:2293. 10.3389/fimmu.2018.02293 30364177 PMC6191518

[B42] Kumkate S. Jenkins G. R. Paveley R. A. Hogg K. G. Mountford A. P. (2007). CD207+ Langerhans cells constitute a minor population of skin-derived antigen-presenting cells in the draining lymph node following exposure to *Schistosoma mansoni*. *Int. J. Parasitol.* 37 209–220. 10.1016/j.ijpara.2006.10.007 17157855 PMC1847335

[B43] Li Z.-Y. Xiao L. Lin G. Tang J. Chen Y. Chen L. (2019). Contribution of tissue transglutaminase to the severity of hepatic fibrosis resulting from *Schistosoma japonicum* infection through the regulation of IL-33/ST2 expression. *Parasit. Vect.* 12:302 10.1186/s13071-019-3542-3544 PMC6570881 31200771

[B44] Magalhães E. S. Paiva C. N. Souza H. S. P. Pyrrho A. S. Mourão-Sá D. Figueiredo R. T. (2009). Macrophage migration inhibitory factor is critical to interleukin-5-driven eosinophilopoiesis and tissue eosinophilia triggered by *Schistosoma mansoni* infection. *FASEB J.* 23 1262–1271. 10.1096/fj.08-124248 19088181

[B45] Magalhães K. G. Almeida P. E. Atella G. C. Maya-Monteiro C. M. Castro-Faria-Neto H. C. Pelajo-Machado M. (2010). Schistosomal-derived lysophosphatidylcholine are involved in eosinophil activation and recruitment through toll-like receptor-2-dependent mechanisms. *J. Infect. Dis.* 202 1369–1379. 10.1086/656477 20863227

[B46] Martinez F. O. Gordon S. (2014). The M1 and M2 paradigm of macrophage activation: time for reassessment. *F1000Prime Rep.* 6:13. 10.12703/P6-13 24669294 PMC3944738

[B47] Mbanefo E. C. Fu C.-L. Ho C. P. Le L. Ishida K. Hammam O. (2020). Interleukin-4 signaling plays a major role in *Urogenital schistosomiasis*-associated bladder pathogenesis. *Infect. Immun.* 88:e00669-19 10.1128/IAI.00669-619 PMC7035943 31843965

[B48] McManus D. P. Dunne D. W. Sacko M. Utzinger J. Vennervald B. J. Zhou X.-N. (2018). Schistosomiasis. *Nat. Rev. Dis. Primers* 4:13 10.1038/s41572-018-0013-18 30093684

[B49] Mountford A. P. Hogg K. G. Coulson P. S. Brombacher F. (2001). Signaling via interleukin-4 receptor alpha chain is required for successful vaccination against *Schistosomiasis* in BALB/c mice. *Infect. Immun.* 69 228–236. 10.1128/IAI.69.1.228-236.2001 11119510 PMC97876

[B50] Mountford A. P. Trottein F. (2004). *Schistosomes* in the skin: a balance between immune priming and regulation. *Trends Parasitol.* 20 221–226. 10.1016/j.pt.2004.03.003 15105022

[B51] Mutengo M. M. Mduluza T. Kelly P. Mwansa J. C. L. Kwenda G. Musonda P. (2018). Low IL-6, IL-10, and TNF-α and High IL-13 cytokine levels are associated with severe hepatic fibrosis in *Schistosoma mansoni* chronically exposed individuals. *J. Parasitol. Res.* 2018:9754060. 10.1155/2018/9754060 29610679 PMC5828471

[B52] Nair M. G. Du Y. Perrigoue J. G. Zaph C. Taylor J. J. Goldschmidt M. (2009). Alternatively activated macrophage-derived RELM-{alpha} is a negative regulator of type 2 inflammation in the lung. *J. Exp. Med.* 206 937–952. 10.1084/jem.20082048 19349464 PMC2715126

[B53] Nascimento M. Huang S. C. Smith A. Everts B. Lam W. Bassity E. (2014). Ly6Chi monocyte recruitment is responsible for Th2 associated host-protective macrophage accumulation in liver inflammation due to *Schistosomiasis*. *PLoS Pathog.* 10:e1004282. 10.1371/journal.ppat.1004282 25144366 PMC4140849

[B54] Nation C. S. Da’dara A. A. Marchant J. K. Skelly P. J. (2020). *Schistosome* migration in the definitive host. *PLoS Negl. Trop. Dis.* 14:7951. 10.1371/journal.pntd.0007951 32240157 PMC7117656

[B55] Paveley R. A. Aynsley S. A. Cook P. C. Turner J. D. Mountford A. P. (2009). Fluorescent imaging of antigen released by a skin-invading helminth reveals differential uptake and activation profiles by antigen presenting cells. *PLoS Negl. Trop. Dis.* 3:e528. 10.1371/journal.pntd.0000528 19829705 PMC2759291

[B56] Paveley R. A. Aynsley S. A. Turner J. D. Bourke C. D. Jenkins S. J. Cook P. C. (2011). The Mannose receptor (CD206) is an important pattern recognition receptor (PRR) in the detection of the infective stage of the helminth *Schistosoma mansoni* and modulates IFNγ production. *Int. J. Parasitol.* 41 1335–1345. 10.1016/j.ijpara.2011.08.005 22036898

[B57] Pearce E. J. Caspar P. Grzych J. M. Lewis F. A. Sher A. (1991). Downregulation of Th1 cytokine production accompanies induction of Th2 responses by a parasitic helminth, *Schistosoma mansoni*. *J. Exp. Med.* 173 159–166. 10.1084/jem.173.1.159 1824635 PMC2118762

[B58] Pearce E. J. MacDonald A. S. (2002). The immunobiology of *Schistosomiasis*. *Nat. Rev. Immunol.* 2 499–511. 10.1038/nri843 12094224

[B59] Peng H. Zhang Q. Li X. Liu Z. Shen J. Sun R. (2016). IL-33 contributes to *Schistosoma japonicum*-induced hepatic pathology through induction of M2 macrophages. *Sci. Rep.* 6:29844. 10.1038/srep29844 27445267 PMC4956744

[B60] Pesce J. T. Ramalingam T. R. Mentink-Kane M. M. Wilson M. S. El Kasmi K. C. Smith A. M. (2009). Arginase-1-expressing macrophages suppress Th2 cytokine-driven inflammation and fibrosis. *PLoS Pathog.* 5:e1000371. 10.1371/journal.ppat.1000371 19360123 PMC2660425

[B61] Prendergast C. T. Sanin D. E. Mountford A. P. (2016). Alternatively activated mononuclear phagocytes from the skin site of infection and the impact of IL-4Rα signalling on CD4+T cell survival in draining lymph nodes after repeated exposure to *Schistosoma mansoni* cercariae. *PLoS Negl. Trop. Dis.* 10:4911. 10.1371/journal.pntd.0004911 27505056 PMC4978413

[B62] Ran L. Yu Q. Zhang S. Xiong F. Cheng J. Yang P. (2015). Cx3cr1 deficiency in mice attenuates hepatic granuloma formation during acute *Schistosomiasis* by enhancing the M2-type polarization of macrophages. *Dis. Model. Mech.* 8 691–700. 10.1242/dmm.018242 26035381 PMC4486856

[B63] Ray D. Nelson T. A. Fu C.-L. Patel S. Gong D. N. Odegaard J. I. (2012). Transcriptional profiling of the bladder in urogenital schistosomiasis reveals pathways of inflammatory fibrosis and urothelial compromise. *PLoS Negl. Trop. Dis.* 6:e1912. 10.1371/journal.pntd.0001912 23209855 PMC3510078

[B64] Rolot M. Dewals B. G. (2018). Macrophage activation and functions during helminth infection: recent advances from the laboratory mouse. *J. Immunol. Res.* 2018:2790627. 10.1155/2018/2790627 30057915 PMC6051086

[B65] Rolot M. Dougall M. A. Javaux J. Lallemand F. Machiels B. Martinive P. (2019). Recruitment of hepatic macrophages from monocytes is independent of IL-4Rα but is associated with ablation of resident macrophages in *Schistosomiasis*. *Eur. J. Immunol.* 49 1067–1081. 10.1002/eji.201847796 30919955

[B66] Rückerl D. Cook P. C. (2019). Macrophages assemble! But do they need IL-4R during *Schistosomiasis*? *Eur. J. Immunol.* 49 996–1000. 10.1002/eji.201948158 31267552 PMC6771897

[B67] Sadler C. H. Rutitzky L. I. Stadecker M. J. Wilson R. A. (2003). IL-10 is crucial for the transition from acute to chronic disease state during infection of mice with *Schistosoma mansoni*. *Eur. J. Immunol.* 33 880–888. 10.1002/eji.200323501 12672053

[B68] Sanin D. E. Mountford A. P. (2015). Sm16, a major component of *Schistosoma mansoni* cercarial excretory/secretory products, prevents macrophage classical activation and delays antigen processing. *Parasit. Vect.* 8:1 10.1186/s13071-014-0608-601 PMC4297449 25561160

[B69] Schistosomiasis (2020). *Schistosomiasis.* Available online at: https://www.who.int/news-room/fact-sheets/detail/schistosomiasis (accessed March 27, 2020).

[B70] Schwartz C. Fallon P. G. (2018). Schistosoma “Eggs-Iting” the host: granuloma formation and egg excretion. *Front. Immunol.* 9:2492. 10.3389/fimmu.2018.02492 30459767 PMC6232930

[B71] Schwartz C. Hams E. Fallon P. G. (2018). Helminth modulation of lung inflammation. *Trends Parasitol.* 34 388–403. 10.1016/j.pt.2017.12.007 29339033

[B72] Shannon P. Markiel A. Ozier O. Baliga N. S. Wang J. T. Ramage D. (2003). Cytoscape: a software environment for integrated models of biomolecular interaction networks. *Genome Res.* 13 2498–2504. 10.1101/gr.1239303 14597658 PMC403769

[B73] Souza C. O. S. Espíndola M. S. Fontanari C. Prado M. K. B. Frantz F. G. Rodrigues V. (2018). CD18 regulates monocyte hematopoiesis and promotes resistance to experimental *Schistosomiasis*. *Front. Immunol.* 9:1970. 10.3389/fimmu.2018.01970 30233576 PMC6127275

[B74] Street M. Coulson P. S. Sadler C. Warnock L. J. McLaughlin D. Bluethmann H. (1999). TNF is essential for the cell-mediated protective immunity induced by the radiation-attenuated schistosome vaccine. *J. Immunol.* 163 4489–4494. 10510391

[B75] Szklarczyk D. Franceschini A. Wyder S. Forslund K. Heller D. Huerta-Cepas J. (2015). STRING v10: protein-protein interaction networks, integrated over the tree of life. *Nucleic Acids Res.* 43 D447–D452. 10.1093/nar/gku1003 25352553 PMC4383874

[B76] Tang H. Liang Y.-B. Chen Z.-B. Du L.-L. Zeng L.-J. Wu J.-G. (2017). Soluble egg antigen activates M2 macrophages via the STAT6 and PI3K Pathways, and *Schistosoma japonicum* alternatively activates macrophage polarization to improve the survival rate of septic mice. *J. Cell. Biochem.* 118 4230–4239. 10.1002/jcb.26073 28419526

[B77] Toffoli da Silva G. Espíndola M. S. Fontanari C. Rosada R. S. Faccioli L. H. Ramos S. G. (2016). 5-lipoxygenase pathway is essential for the control of granuloma extension induced by Schistosoma mansoni eggs in lung. *Exp. Parasitol.* 167 124–129. 10.1016/j.exppara.2016.06.001 27262746

[B78] Torben W. Ahmad G. Zhang W. Nash S. Le L. Karmakar S. (2012). Role of antibody dependent cell mediated cytotoxicity (ADCC) in Sm-p80-mediated protection against *Schistosoma mansoni*. *Vaccine* 30 6753–6758. 10.1016/j.vaccine.2012.09.026 23000221 PMC3488153

[B79] Turner J. D. Bourke C. D. Meurs L. Mbow M. Dièye T. N. Mboup S. (2014). Circulating CD14brightCD16+ “intermediate” monocytes exhibit enhanced parasite pattern recognition in human helminth infection. *PLoS Negl. Trop. Dis.* 8:e2817. 10.1371/journal.pntd.0002817 24762736 PMC3998941

[B80] Vannella K. M. Ramalingam T. R. Borthwick L. A. Barron L. Hart K. M. Thompson R. W. (2016). Combinatorial targeting of TSLP, IL-25, and IL-33 in type 2 cytokine-driven inflammation and fibrosis. *Sci. Transl. Med.* 8:337ra65. 10.1126/scitranslmed.aaf1938 27147589

[B81] Varol C. Landsman L. Fogg D. K. Greenshtein L. Gildor B. Margalit R. (2007). Monocytes give rise to mucosal, but not splenic, conventional dendritic cells. *J. Exp. Med.* 204, 171–180. 10.1084/jem.20061011 17190836 PMC2118434

[B82] von Lichtenberg F. Sher A. McIntyre S. (1977). A lung model of schistosome immunity in mice. *Am. J. Pathol.* 87 105–123. 851162 PMC2032075

[B83] Wilson R. A. (2009). The saga of schistosome migration and attrition. *Parasitology* 136 1581–1592. 10.1017/S0031182009005708 19265564

[B84] Winkel B. M. F. Dalenberg M. R. de Korne C. M. Feijt C. Langenberg M. C. C. Pelgrom L. (2018). Early Induction of human regulatory dermal antigen presenting cells by skin-penetrating *Schistosoma mansoni* Cercariae. *Front. Immunol.* 9:2510. 10.3389/fimmu.2018.02510 30429854 PMC6220649

[B85] Wolde M. Laan L. C. Medhin G. Gadissa E. Berhe N. Tsegaye A. (2020). Human monocytes/macrophage inflammatory cytokine changes following in vivo and in vitro *Schistomam manoni* infection. *J. Inflamm. Res.* 13 35–43. 10.2147/JIR.S233381 32021377 PMC6970607

[B86] World Health Organization [WHO] (2020). *Epidemiological Situation.* Geneva: WHO.

[B87] Ye Z. Huang S. Zhang Y. Mei X. Zheng H. Li M. (2020). Galectins, eosinophiles, and macrophages may contribute to *Schistosoma japonicum* Egg-induced immunopathology in a mouse model. *Front. Immunol.* 11:146. 10.3389/fimmu.2020.00146 32231658 PMC7082360

[B88] Yong L. Tang Y. Ren C. Liu M. Shen J. Hou X. (2019). B1 cells protect against *Schistosoma japonicum*-induced liver inflammation and fibrosis by controlling monocyte infiltration. *PLoS Negl. Trop. Dis.* 13:e0007474. 10.1371/journal.pntd.0007474 31194740 PMC6592576

[B89] Yu Y. Deng W. Lei J. (2015). Interleukin-33 promotes Th2 immune responses in infected mice with *Schistosoma japonicum*. *Parasitol. Res.* 114 2911–2918. 10.1007/s00436-015-4492-4491 25944738

[B90] Zhao Y. Zou W. Du J. Zhao Y. (2018). The origins and homeostasis of monocytes and tissue-resident macrophages in physiological situation. *J. Cell. Physiol.* 233 6425–6439. 10.1002/jcp.26461 29323706

[B91] Zhu J. Xu Z. Chen X. Zhou S. Zhang W. Chi Y. (2014). Parasitic antigens alter macrophage polarization during *Schistosoma japonicum* infection in mice. *Parasit. Vect.* 7:122. 10.1186/1756-3305-7-122 24666892 PMC3975460

